# Performance of Children With Johanson-Blizzard Syndrome After Cochlear Implantation

**DOI:** 10.7759/cureus.19264

**Published:** 2021-11-04

**Authors:** Rawan M Alwadee, Mohammed Y Alyousef, Einas M Yousef, Medhat F Yousef

**Affiliations:** 1 Department of Otolaryngology, College of Medicine, King Saud University, Riyadh, SAU; 2 Basic Medical Sciences, College of Medicine, Dar Al Uloom University, Riyadh, SAU; 3 Histology and Cell Biology, Faculty of Medicine, Menoufia University, Sheben ElKom, EGY; 4 Audiology Unit, ENT Department, Faculty of Medicine, Menoufia University, Sheben ElKom, EGY; 5 Audiology Unit, King Abdullah Ear Specialist Centre (KAESC), College of Medicine, King Saud University, Riyadh, SAU

**Keywords:** sir, cap, ubr1 gene, cochlear implantation, sensorineural hearing loss, johanson-blizzard syndrome

## Abstract

Johanson-Blizzard syndrome (JBS) is a rare autosomal recessive hereditary disorder characterized by multi-system involvement and facial dysmorphic features. One of the most common symptoms in JBS patients is bilateral severe to profound sensorineural hearing loss. The objective of this report is to highlight the performance of those patients after receiving cochlear implants (CI) as a management for their hearing loss. In this study, we reviewed the medical records of one female child diagnosed with JBS before and after cochlear implantation, with a particular focus on their auditory and language performance. After receiving the cochlear implant, our patient showed substantial improvement in her hearing threshold and communication abilities when compared to the preoperative condition. In conclusion, although cochlear implantation is considered a good approach for the management of JBS patients, the development of spoken language is not always achieved.

## Introduction

Johanson-Blizzard syndrome (JBS) (OMIM: #243800) is a rare autosomal recessive disorder first described in 1971 by Johanson and Blizzard [1]. It is characterized by developmental delay and several dysmorphic features such as microcephaly, absence of permanent teeth, hypoplasia of the alae nasi, and scalp defects. Other characteristic features include exocrine pancreatic insufficiency, urogenital malformations, hypothyroidism, congenital heart defects, and sensorineural hearing loss [2]. JBS is caused by homozygous and compound heterozygous mutations of the UBR1 gene (MIM *605981), encoding a ubiquitin ligase of the N-end rule pathway which is involved in ubiquitin-mediated degradation of various proteins [3,4]. The prevalence of JBS is estimated to be 1 in 250,000, with no reported difference in gender while parental consanguinity is frequently observed [5].

To our knowledge, only two studies reported the outcomes of cochlear implantation with JBS patients, however one of them was in Russian [6,7]. The objective of the current case report is to present the auditory performance, speech and language development of a seven-year-old female diagnosed with JBS with a bilateral sensorineural hearing loss (SNHL) who underwent a unilateral left cochlear implantation.

## Case presentation

A seven-year-old female with JBS presented to our clinic for evaluation of the possibility of cochlear implantation for bilateral SNHL. She was born in the USA to healthy first-cousin parents with no family history of any major or genetic disorders. She was found to have facial dysmorphic features such as hypertelorism, a beaked nose, pinpoint chin, low hairline, wide spaced small nipples, and café au lait spots distributed over her body. She was diagnosed with multiple conditions known to be associated with JBS such as pancreatic insufficiency, patent ductus arteriosus with left to right shunt, recto-vestibular fistula, tethered cord, and global developmental delay.

**Figure 1:**
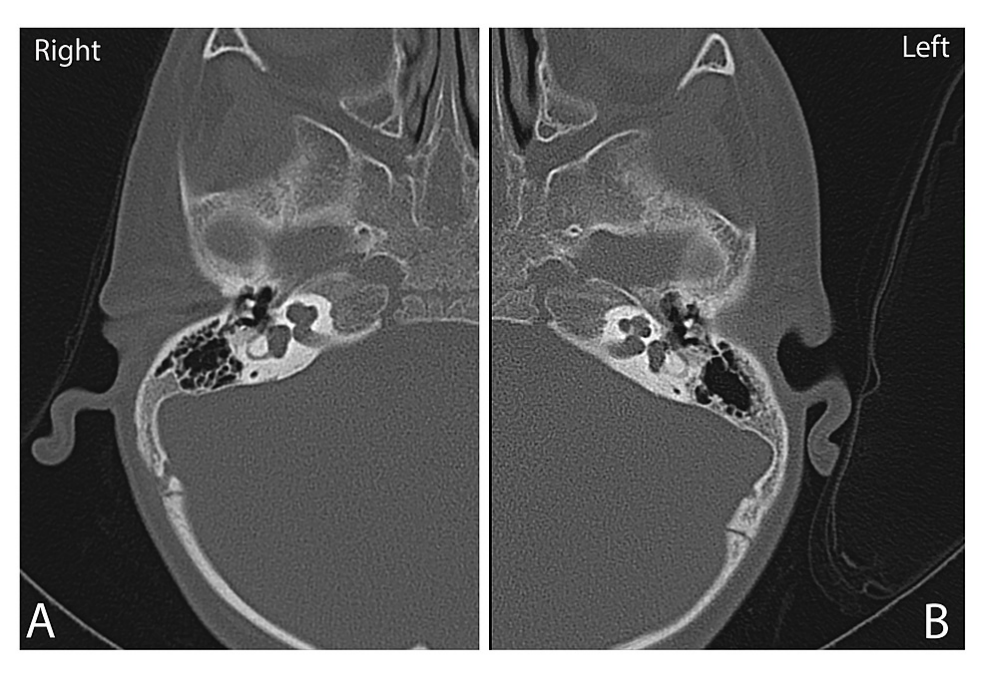
Computed tomography (CT) scan of the inner ears A) CT scan of the right ear shows right cochlear anomaly with incomplete partition type II and enlarged vestibule B) CT scan of left inner ear shows normal appearance of cochlea and modiolus.

Our patient failed the newborn hearing screening test bilaterally. At the age of three months, her hearing evaluation using auditory brainstem response revealed bilateral severe to profound SNHL. At the age of five months, she was fitted with bilateral hearing aids. After one year of follow up, her parents reported limited aided responses. They also reported that she was not reacting except to loud sounds along with speech delay. Moreover, her mode of communication was mainly by pointing and responding to simple commands, and that she has normal playing skills. At the age of three years, she was referred to the cochlear implantation (CI) committee in our hospital for evaluation. Radiological evaluations revealed right cochlear anomaly with incomplete partition type II of the right middle and apical cochlear turns with enlarged vestibule, absent modiolus, and hypoplastic cochlear nerve (Figure 1). It also showed normal left cochlea and cochlear nerve. As for her Stanford Binet Intelligence Scales "Fifth Edition" score, it was 95 (91-99) which is classified as average IQ. Based on these investigations, she was accepted by the hospital CI committee for unilateral left cochlear implantation. 

At four years of age, she received a left CI (Synchrony-FORM24; MedEl, Innsbruck, Austria) with complete insertion of the electrode array. Intra-operative objective assessment revealed normal impedances and presence of electric compound action potential (ECAP) responses from all electrodes. There were no recorded postoperative complications. Following the implantation, the patient was regularly brought to her follow-up appointments and rehabilitation clinic. She showed improvement with the rehabilitation process. Her most recent speech detection threshold (SDT) was 35 decibel (dB) hearing loss (HL) and the aided hearing threshold ranged between 35 and 40 dB HL (Table 1). After three years of implantation, an integrated scale of development tool was used to assess her progress in the six key areas of development, relative to hearing and chronological ages [8]. While at the time of evaluation her hearing age was 36 months, her scores were as following: audition: 13-15 months, receptive language: 13-15 months, expressive language: 10-12 months, cognitive: 31-36 months (emerge), speech: 10-12 months. Categories of Auditory Performance (CAP) score was 3/9 which means that she can recognize the environmental sounds [9]. In addition, her speech intelligibility rating (SIR) score was 1, which indicated that her connected speech is unintelligible and her primary mode of communication may be manual [10]. The speech therapist reported that she depends on lip reading and she is using gestures for communication. She attended a governmental special educational needs school.

**Table 1:** Hearing threshold of left ear before and after left ear implantation

	250 Hz	500 Hz	1000 Hz	2000 Hz	4000 Hz	8000 Hz
Before implantation	80	90	95	100	NR	NR
After implantation	35	40	40	35	35	40

## Discussion

JBS is a rare autosomal recessive condition caused by mutations in the gene encoding UBR1. Our patient presented with multi-system involvement, delayed global development, and average intellectual disability. Our findings are consistent with other studies which reported a very similar picture of JBS [2]. However, two other different studies reported cases of JBS with normal intelligence [11,12]. The variation in the phenotypic features of those patients can be explained by having different types of mutations that affect the gene encoding UBR1. To our knowledge, only two studies reported the outcomes of cochlear implantation with JBS patients, however one of them was in Russian [6,7].

One of the main problems in our case was the severe to profound SNHL which negatively impacted her communication abilities. Unfortunately, our patient did not benefit from hearing aids which made her a good candidate for CI. The results of radiological evaluation revealed congenital anomaly of the right cochlea and normal left inner ear. Hence, unilateral left ear implantation was conducted for this patient. Our patient showed substantial improvement in her hearing threshold and communication abilities when compared to the preoperative condition (Table 1). These results showed how CI gave her good access and audibility to different sounds in the speech spectrum which helped her to get more benefit from the special educational needs school. However, her integrated scales of development three years after the implantation showed a delay in the six key areas of development (audition: 13-15 months, receptive language: 13-15 months, expressive language: 10-12 months, cognitive: 31-36 months (emerge), speech: 10-12 months) in comparison to her hearing and chronological ages. Furthermore, her CAP score was 3/9 which indicates that she can recognize the environmental sounds; while her SIR score was 1/5 which indicates that her connected speech is unintelligible, and her primary mode of communication may be manual. These results were consistent with the findings of a previous case report conducted by Holcomb et al. who reported a comparative degree of improvement in both CAP and SIR scores. Although their patient had great difficulty with processing spoken language, he showed improvement in his communication with sign language, and he was able to respond to speech stimuli and environmental sounds using his cochlear implants [6]. The observed delay in the development scales can be attributed to the delayed referral of the patient for the CI committee which resulted in late implantation, multiple system affection, delayed global development, and average intellectual disability, all of which had a detrimental effect on her language development. Additionally, the coronavirus disease 2019 (COVID-19) scenario had a significant impact on the face-to-face rehabilitation sessions and attending the school.

## Conclusions

In general, early diagnosis of JBS syndrome can improve patient outcomes through guiding variable treatment interventions. Early audiological evaluation is recommended for those patients to diagnose any hearing impairment as early as possible, to maximize the good outcome of the cochlear implantation. In our opinion, early and bilateral CI is highly recommended as management of SNHL of JBS. Additionally, prenatal genetic counseling is critical, particularly in cases of consanguineous marriage.

Our findings indicate that, while cochlear implantation is a viable option for managing JBS patients with bilateral SNHL, other factors such as the patient's intellectual disability and other systems affection can significantly affect postoperative outcomes, as spoken language development is not always achieved. The current data support the use of early CI in JBS patients. Multidisciplinary care, some educational services for hearing impairment, and specialist counseling tactics for behavioral manifestations following CI may all contribute to improving the quality of life for JBS patients.
